# Idiopathic true ulnar artery aneurysm

**DOI:** 10.1016/j.ijscr.2021.105821

**Published:** 2021-03-23

**Authors:** Yusuf Khaled Abdulghaffar Abdulla, Dhafer M. Kamal, Keith Pappachen Mathew

**Affiliations:** a Bahrain Defence Force Hospital-Royal Medical Services, Riffa, Bahrain; b Royal College of Surgeons in Ireland University, Bahrain

**Keywords:** Ulnar artery, True aneurysm, Idiopathic, Case report

## Abstract

**Introduction and importance:**

True ulnar artery aneurysms are a rare entity, with existing literature suggesting that most of these aneurysms are due to trauma. This case report sheds light on a true ulnar artery aneurysm that was deemed to be idiopathic.

**Case presentation:**

A 49-year-old lady presented with numbness in the medial 3 fingers of the left hand, tenderness over the medial forearm, and a palpable ulnar artery pulse. There was no history of trauma. An arterial ultrasound, MRI of the forearm, and upper limb angiography confirmed the diagnosis of ulnar artery aneurysm. Surgical repair of the aneurysm was done with excellent immediate post-operative and follow up results.

**Clinical discussion:**

Trauma is the most common cause for the development of ulnar artery aneurysms. In this case however, after detailed history taking, physical examination, and thorough investigations, the cause of the patient’s aneurysm was deemed to be idiopathic. Treatment of ulnar aneurysms are not well established due to the rarity of the disease. A surgical approach however may be considered depending on the location of the aneurysm along with the patient’s symptoms.

**Conclusion:**

A high index of suspicion, thorough history, physical examination, and appropriate investigations are required to diagnose ulnar artery aneurysms. Surgical intervention is indicated to relieve symptoms of nerve compression and prevent limb threatening ischemia. Regular post-operative follow up is essential to detect potential graft dysfunction and to ensure a good outcome.

## Highlights

• Idiopathic true ulnar artery aneurysms are rare.
• Diagnosis of idiopathic ulnar artery aneurysms is by thorough history-taking, examination, and other imaging modalities.
• Treatment of idiopathic ulnar artery aneurysm depends on its location and associated symptoms.

## 1 Introduction

An aneurysm is an abnormal focal dilation of a blood vessel that exceeds the normal diameter by more than 50% due to weakness of the vascular wall. Aneurysms may occur in any blood vessel but are more often seen in arteries rather than veins [1]. A true aneurysm involves all three layers of the blood vessel, including the tunica intima, media, and adventitia [2].

Aneurysms can occur in many arteries of the body, the most commonly involved are the aorta, cerebral vessels, and popliteal artery. Ulnar aneurysms however, are a rare entity, with existing literature suggesting that the cause of such aneurysm is trauma [3]. Injuries to the ulnar artery may be secondary to repeatedly striking the palmar aspect of the medial hand and wrist against objects as seen in certain occupations and minor paediatric fall injuries. Other causes are attributed to infection, vasculitis, connective tissue diseases and quite rarely, idiopathic.

This case aims to present a unique medical occurrence of an idiopathic true ulnar artery aneurysm in a middle-aged female. The following work has been reported in line with the SCARE 2020 criteria [12].

## 2 Case presentation

A forty-nine-year-old female who was not a known case of any medical illnesses, presented to the outpatient clinic with a two-week history of pain in the left forearm, and numbness over the 3rd, 4th and 5th digits and medial aspect of the hand, which limited her daily living activities. This was associated with pallor of the ulnar aspect of the hand during excessive use. The patient denied having loss of motor functioning and trauma to the affected limb. Moreover, the patient did not have any risk factors for aneurysmal formation such as smoking, hypertension, dyslipidaemia, or connective tissue disease. She did not have any cardiovascular complains either.

General examination was normal. A peripheral vascular exam of both upper limbs showed no skin changes, ulcers, or peripheral cyanosis. Skin temperature and capillary refill time was normal. All upper limb pulses were present bilaterally, however, the left distal ulnar artery pulse was faint, whilst the pulse at the aneurysm’s site was prominent. Tenderness was present in the medial left forearm. Sensation was reduced over the left 3rd, 4th and 5th digits suggesting an ulnar nerve distribution. Cardiovascular examination was unremarkable.

**Fig. 1:**
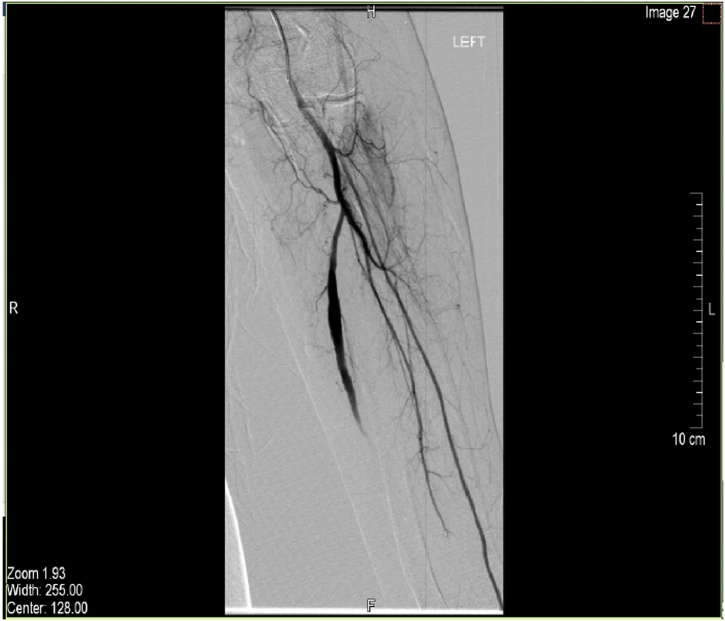
Upper limb angiography showing aneurysmal dilatation of the proximal part of the ulnar artery.

**Fig. 2:**
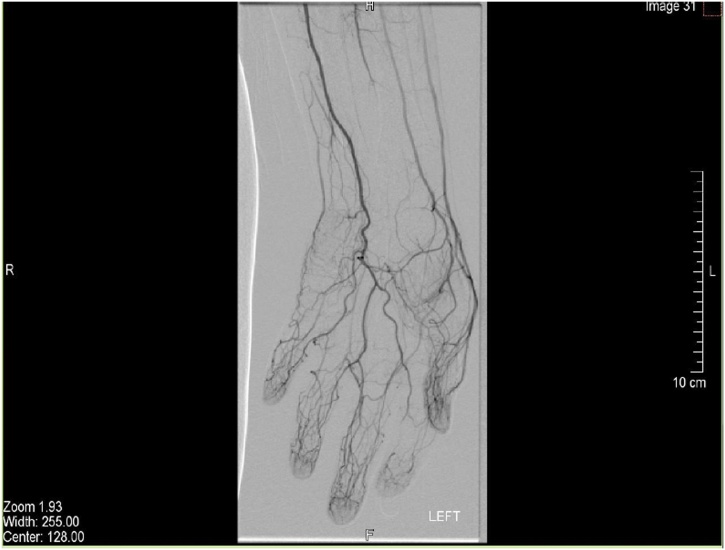
Hand angiography showing discontinuous palmar arch.

**Fig. 3:**
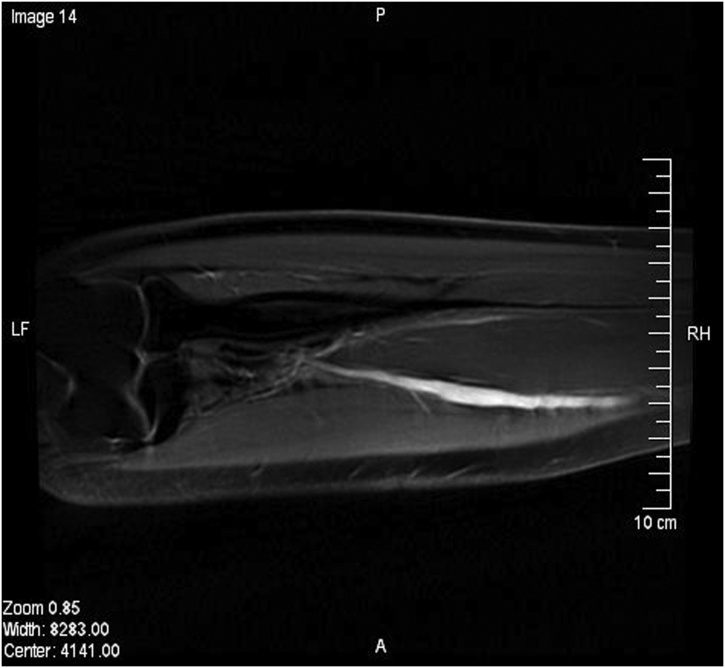
Longitudinal MRI of the forearm showing ulnar artery aneurysm.

**Fig. 4:**
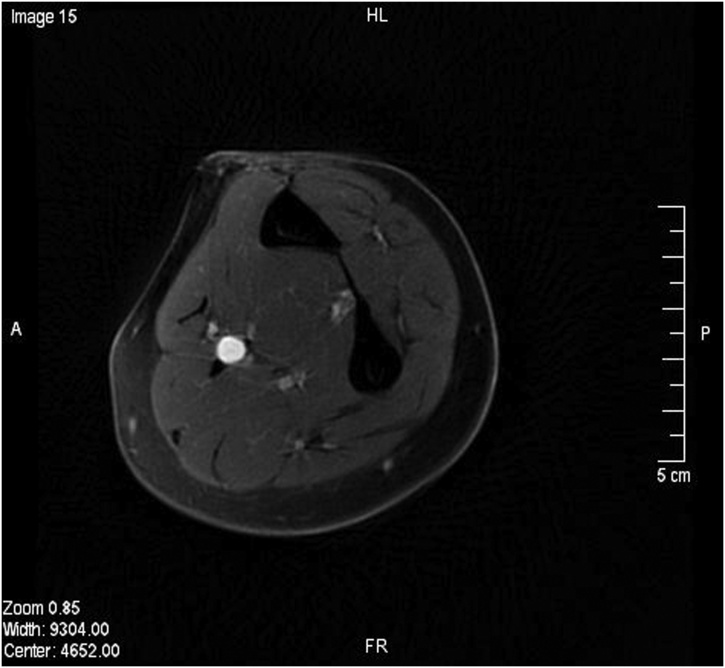
Axial MRI of the forearm showing ulnar artery aneurysm.

Blood tests for vasculitis and connective tissue disorders were sent and results were reported as negative. A duplex ultrasound of the left forearm showed a dilated middle segment of the ulnar artery with limited flow. These findings were confirmed with an MRI (Fig. 3, Fig. 4). Subsequently, a left upper limb angiography revealed a fusiform aneurysm of the proximal to mid segment of the left ulnar artery measuring approximately 9 cm in length and 8 mm in diameter (Fig. 1). A sluggish flow was noted in the ulnar artery in comparison to the radial and interosseous arteries. The aortic arch, subclavian, axillary, brachial and radial arteries were patent with no evidence of atherosclerotic changes. The palmar arch was discontinuous; with the radial artery supplying the radial two-and-a-half digits, and the ulnar artery supplying the ulnar two-and-a-half digits (Fig. 2). The ulnar artery was anatomically variant. Angiography revealed that the patient’s ulnar artery aneurysm was double the size of a regularly functioning ulnar artery. As the ulnar artery lies adjacent to the ulnar nerve, the clinical presentation and radiological findings of this patient suggested symptomatic ulnar artery aneurysm due to ulnar nerve compression. Therefore, a decision was made to repair the aneurysm.

A left ulnar artery aneurysm repair was performed by the senior consultant of vascular surgery. First, an antecubital fossa incision was made, identifying the distal brachial artery and both ulnar and radial arteries. The ulnar artery was dissected out distally until it went deep into the muscular planes of the medial forearm. A distal medial forearm incision was made, and tissue dissection was carried out until the ulnar artery and nerve were identified and preserved. The aneurysmal portion of the ulnar artery was seen, and further dissection was carried out to the healthy portion of the artery distally. A subcutaneous tunnel was made between the two incisions. A below-the-knee portion of the right great saphenous vein was harvested in the usual fashion and passed through the tunnel made in the forearm in a reversed fashion. The vessels were clamped after systemic heparinization. Prolene 6.0 and 7.0 sutures were used to perform the proximal and the distal anastomoses, respectively. A full thickness layer of the distal part of the aneurysm was excised and sent for histopathological examination. Post-operatively, good left ulnar and radial artery pulses were felt, and the graft was functioning well.

The histopathology report revealed a true aneurysm of the ulnar artery with no signs of inflammation. The patient’s hospital stay was uneventful, and she was discharged home on the 4th post-operative day.

**Fig. 5:**
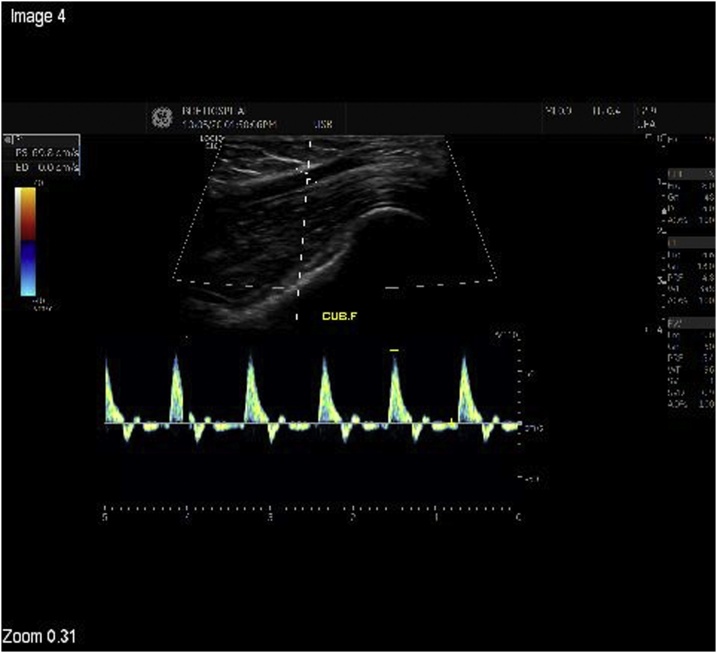
Follow up graft duplex ultrasound showing satisfactory flow without evidence of stenosis at the proximal or distal anastomoses.

The patient was followed up regularly in the surgical clinic. She was doing well, apart from having initial occasional numbness of the little finger and half of ring finger which resolved on further follow up. All incisions healed well and both radial and ulnar pulses were easily palpable. A duplex ultrasound was done to assess the patency of the bypass, proving to be patent with good flow at 3, 6, 9, 12, 18, 24, and 30 months post-operatively (Fig. 5). Currently the patient remains asymptomatic.

## 3 Discussion

Ulnar artery aneurysms are a rare entity. Those that occur, are often caused by trauma, vasculitis, connective tissue diseases or are iatrogenic. In this case however, after a detailed history, clinical examination, and thorough investigations were done, the cause of the patient’s ulnar artery aneurysm was deemed to be idiopathic.

A study by S Roshan Rodney et al. also diagnosed an idiopathic non-traumatic ulnar aneurysm in a twenty-nine-year-old male. Their patient presented with a gradually increasing right hand and forearm pain for four days associated with a noticeable lump over the distal ulnar aspect [4]. On examination, the radial pulse was felt but the ulnar artery pulse was absent [4]. This suggests that idiopathic ulnar arteries may present differently as seen in this case in comparison to our case.

In Nickul N Shah report [5], the patient experienced numbness and tingling sensation in the 4th and 5th digits as did our patient, which indicates ulnar nerve compression secondary to ulnar artery aneurysm in both cases.

Traumatic ulnar artery aneurysms may occur due to repetitive trauma to the hypothenar eminence in athletes and certain occupations. This may cause hypothenar hammer syndrome [6]. Another ulnar artery aneurysm was reported by B. Kisacik, in a patient with Behcet’s disease who presented with a sudden onset of swelling of the left hypothenar eminence [7]. The inflammatory element of Behcet’s disease led to aneurysm formation [8].

Most non-distal ulnar artery aneurysms are found in children. Mehmet Erdem reported a five-year-old girl with a proximal ulnar artery aneurysm, that was asymptomatic, and found incidentally as a pulsatile mass on examination [9].

Treatment of ulnar aneurysms is not well established due to the rarity of the disease. The decision of surgical intervention depends on the aneurysm’s location and the patient’s symptoms. If the aneurysm is distal, simple resection is a suitable option if the hand is adequately perfused with an intact radial artery [10]. However, if hand perfusion is inadequate, ulnar artery reconstruction using microsurgical technique is mandatory [11]. The reconstruction can be achieved by primary end-to-end anastomosis if there is no tension. In our case, however, the aneurysm was in the proximal to middle segment of the ulnar artery, with features suggesting ulnar nerve compression. Moreover, if left untreated, the aneurysm may form a thrombus which may lead to distal embolization, critical ischemia, and limb loss. In addition, the patient had features suggesting ulnar nerve compression. Therefore, surgical repair was sought. A percutaneous endovascular repair using a stent-graft is an attractive option. However, data on long-term results in such case is lacking. We therefore, repaired the aneurysm surgically with an interposition vein graft tunnelled subcutaneously since the bulk of the aneurysm was located deep to the flexor muscles of the forearm. Post-operatively, the patient’s symptoms and signs resolved, and excellent graft function and patency rate was achieved as documented by ultrasound at regular intervals up until the time of writing this paper (30 months post-operatively).

## 4 Conclusion

Ulnar artery aneurysms are rare and are often secondary to trauma. They may however, be idiopathic. A focused history and examination followed by appropriate investigations and radiological imaging are therefore important to attain the diagnosis. Such aneurysms must be repaired to treat nerve compression and prevent potential limb threatening ischemia. Despite the various methods available for surgical repair, approach must be guided by presentation, patient comorbidities and the surgeon’s expertise. Timely follow-up at regular intervals with thorough examination and diagnostic modalities is important to identify potential post-operative complications and ensure a good prognosis.

## Patient perspective

The patient was asked to share her perspective on the treatment she received for her condition. The following is what the patient had to say:

“After my operation, I remained to have numbness over my little and ring finger, which quite frankly didn’t last for long. I am currently symptom free and have no issues in regards to this matter. Thank you for relieving me from my symptoms.”

## Declaration of Competing Interest

None declared.

## Funding

None declared.

## Ethical approval

Approval from the Bahrain Defence Force Royal Medical Services Research & Research Ethics Committee has been attained. Reference number: 2020-498.

## Consent

Written informed consent was obtained from the patient for publication of this case report and accompanying images. A copy of the written consent is available for review by the Editor-in-Chief of this journal on request.

## Author contribution

Dr. Yusuf Khaled Abdulghaffar Abdulla (corresponding author) – writing the paper and data collection.

Dr. Dhafer Kamal (supervisor) – study concept and data analysis.

Dr. Keith Pappachen Mathew – contributing to writing the paper.

## Registration of research studies

Not applicable.

## Guarantor

Dr. Yusuf Khaled Abdulghaffar Abdulla.

## Prevenance and peer review

Not commissioned, externally peer-reviewed.
